# Single-cell analysis of the adaptive immune response to SARS-CoV-2 infection and vaccination

**DOI:** 10.3389/fimmu.2022.964976

**Published:** 2022-09-02

**Authors:** Furong Qi, Yingyin Cao, Shuye Zhang, Zheng Zhang

**Affiliations:** ^1^ Institute for Hepatology, National Clinical Research Center for Infectious Disease, Shenzhen Third People’s Hospital, The Second Affiliated Hospital, School of Medicine, Southern University of Science and Technology, Shenzhen, China; ^2^ Shenzhen Key Laboratory of Single-Cell Omics Reasearch and Application, Shenzhen, China; ^3^ Clinical Center for BioTherapy and Institutes of Biomedical Sciences, Zhongshan Hospital, Fudan University, Shanghai, China; ^4^ Shenzhen Research Center for Communicable Disease Diagnosis and Treatment of Chinese Academy of Medical Science, Shenzhen, China

**Keywords:** SARS-CoV-2, infection, vaccine, adaptive immune response, antibody production

## Abstract

Amid the ongoing Coronavirus Disease 2019 (COVID-19) pandemic, vaccination and early therapeutic interventions are the most effective means to combat and control the severity of the disease. Host immune responses to SARS-CoV-2 and its variants, particularly adaptive immune responses, should be fully understood to develop improved strategies to implement these measures. Single-cell multi-omic technologies, including flow cytometry, single-cell transcriptomics, and single-cell T-cell receptor (TCR) and B-cell receptor (BCR) profiling, offer a better solution to examine the protective or pathological immune responses and molecular mechanisms associated with SARS-CoV-2 infection, thus providing crucial support for the development of vaccines and therapeutics for COVID-19. Recent reviews have revealed the overall immune landscape of natural SARS-CoV-2 infection, and this review will focus on adaptive immune responses (including T cells and B cells) to SARS-CoV-2 revealed by single-cell multi-omics technologies. In addition, we explore how the single-cell analyses disclose the critical components of immune protection and pathogenesis during SARS-CoV-2 infection through the comparison between the adaptive immune responses induced by natural infection and by vaccination.

## Introduction

Single-cell (sc) technologies, including flow cytometry (FACS), mass cytometry (CyTOF), cellular indexing of transcriptomes and epitopes by sequencing (CITE-seq), single-cell RNA sequencing (scRNA-seq), single-cell assay for transposase-accessible chromatin using sequencing (scATAC-seq), single-cell T cell receptor sequencing (scTCR-seq), and single-cell B cell receptor sequencing (scBCR-seq), are well reviewed to characterize the heterogeneity of innate or adaptive immune responses to vaccination, infection, and cancer (1, 2). Briefly, they can 1) address immune heterogeneity and identify novel cell subsets; 2) offer more accurate descriptions of cell phenotypes and their responses to vaccination and infection; 3) deduce the transition, differentiation or developmental relationships between cell subsets; 4) explore the function of antigen-specific cells; 5) characterize the immune repertoire such as functional TCRs and BCRs. These single-cell technologies have been extensively adopted to study the protective and pathogenic mechanisms and to develop new strategies to prevent and treat COVID-19 (3–8).

Especially, the antigen-specific adaptive immune responses, which generally play a vital role in controlling most viral infections (9), are revealed by single-cell technologies in SARS-CoV-2 infection and vaccination. This role is reflected in its critical impact on the various clinical outcomes of SARS-CoV-2 infection and on the efficacy of vaccination. B cells, CD4^+^ T cells, and CD8^+^ T cells are three critical arms of the adaptive immune system, contributing to the control of SARS-CoV-2 and the development of immune memory (10–12). CD4^+^ T cells perform effector functions and provide assistance for other immune cells (13), while CD8^+^ T cells are important tissue-resident lymphocytes which are ready to eradicate virus-infected cells (14). B cells mainly produce antibodies to neutralize viruses or target virus-infected cells in an Fc-mediated manner (15). Both SARS-CoV-2 infection and vaccination effectively elicited T cell and B cell responses against SARS-CoV-2 (3, 16), providing a high level of protection against symptomatic SARS-CoV-2 infection (17), despite the challenges posed to the waned immunity by the immunity-escaping emerging viral variants. Of note, single-cell analysis together with the classic immunological assay, have greatly contributed to deeper understanding of the dynamics of SARS-CoV-2-specific CD4^+^ and CD8^+^ T cells, B cells and neutralizing antibodies (18–23) during natural infection and various vaccination (**Table 1**) (24–36). A comprehensive comparison of the adaptive immune responses with natural infection and vaccination is required to dissect the protective immune properties against SARS-CoV-2 infection. Here, we will review the current knowledge of SARS-CoV-2-specific immune responses during natural infection or vaccination and highlight future directions.

**Table 1:** The approved COVID-19 vaccines explored by single-cell technologies.

Vaccines	Vaccine type	Immunogens	Reference
mRNA-1273	mRNA	Spike protein	(24–26)
BTN162b2	mRNA	Spike protein	(24, 27–33)
AZD1222	Non-replicating viral vector	Spike protein	(32)
Ad5-nCoV	Non-replicating viral vector	Spike protein	(34)
BBIBP-CorV	Inactivated SARS-CoV-2 virus	Multiple viral antigens	(35)
CoronaVac	Inactivated SARS-CoV-2 virus	Multiple viral antigens	(36)

### T cell response against SARS-CoV-2

Studies have shown that both SARS-CoV-2 infection and vaccination elicited robust anti-viral T cell responses, showing an increased expression of T-cell relevant cytotoxic signatures (3, 16). A variety of CD4^+^ and CD8^+^ T cell subsets were characterized by scRNA-seq analyses. The reported CD4^+^ T cell subsets by scRNA-seq mainly include naïve (*CCR7* ^+^, *TCF7^high^*, *LEF1^high^*), T-helper 1 like cells (T_H_1, *TBX21* ^+^ *GNLY* ^+^), T-helper 2 like cells (T_H_2, *GZMK* ^+^ *GATA2* ^+^), proliferative cells (*MKI67* ^+^), T follicular helper cells (T_FH_, *ICOS* ^+^), T-helper 17 like cells (T_H_17, *RORC* ^+^ *CCR6* ^+^), T-regulatory cells (Treg, *CTLA4* ^+^ *FOXP3* ^+^), and interferon-stimulated genes (ISGs^high^) cells. The reported CD8^+^ T cell subsets by scRNA-seq mainly included naïve (*CCR7* ^+^), effector memory (T_EM_, *GZMK* ^+^ *LTB* ^+^), cytotoxic terminal effector (T_TE_, *GNLY* ^+^ *GZMB* ^+^ *PRF1* ^+^), exhausted-like (T_EX_, *PDCD1* ^+^ *LAG3* ^+^), tissue-resident memory (T_RM_, *ITGAE* ^+^ *CXCR6* ^+^ *ZNF683* ^+^), and proliferative (*MKI67* ^+^) cells (4, 5, 37–53) (**Table 2**). The antigen specificity, cross-reactivity, composition and transcription features of these T cell subsets have been extensively explored.

**Table 2:** The T cell and B cell subsets identified by single cell technologies and their canonical markers.

Cell subsets	Trait	scRNA-seq marker	Flow cytometry/Mass spectrometry marker	Reference
CD4^+^ T cell subsets	T_H_1-like	*GZMB, GNLY, PRF1, TBX21*	CD45RA^-^, CXCR5^-^, CCR6^-^, CXCR3^+^, CCR4^-^	(4, 42–45)
	T_H_2-like	*GATA3, GZMK*	CD45RA^-^, CXCR5^-^, CCR6^-^, CXCR3^-^, CCR4^+^	(4, 45, 46)
	T_H_17-like	*CCR6, RORC*	CD45RA^-^, CXCR5^-^, CCR6^+^	(4, 5)
	T_FH_	*ICOS, CXCR5*	CD45RA^-^, CXCR5^+^	(4, 38)
	Treg	*FOXP3, CTLA4*	CD25^+^, CD127^-^	(4, 38, 44)
	Naive	*CCR7, SELL, TCF7*	CD45RA^+^, CD62L^+^	(4, 43–45)
	Proliferative	*MKI67, TOP2A*	Ki67^+^	(4, 6)
	ISGs+ subsets	*ISG15, ISG20, IFI6*	-	(4, 16)
CD8^+^ T cell subsets	Naive	*CCR7, SELL, TCF7*	CD45RA^+^, CD62L^+^, CD95^-^	(43–45)
	T_TE_	*GZMB, GNLY, PRF1*	CD244^+^, KLRG1^+^	(43–45)
	T_EM_	*GZMK, LTB*	CD45RA^−^, CD127^+^, CD28^+^, CD95^+^	(40, 43–45)
	T_RM_	*CXCR6, ITGAE, ZNF683*	CXCR6^+^, CD103^+^	(5, 40, 41)
	T_EX_	*PDCD1, LAG3*	PD-1^+^	(40, 43, 47)
	Proliferative	*MKI67, TOP2A*	Ki67^+^	(4, 6, 43)
	ISGs+ subsets	*ISG15, ISG20, IFI6*	-	(6, 16)
B cell subsets	Naive	*TCL1A, BACH2*	IgD^+^, CD27^-^	(4, 45, 48, 49)
	Class-switched MBC	*IGHD^-^, CD27^+^*	IgD^-^, CD27^+^	(42, 45, 49, 50)
	Not-class-switched MBC	*IGHD^+^, CD27^+^*	IgD^+^, CD27^+^	(42, 49)
	Plasma cells	*MZB1, CD38, SDC1*	CD27^+^, CD38^+^	(42, 48, 49)
	Plasmablasts	*MZB1, CD38, MKI67*	Ki67^+^	(42, 45, 48, 49)
	Immature B cell	*IGHM, MME*	CD20^+^, CD24^+^, CD38^+^	(49, 51)
	Exhausted B cell	*CD27^-^, CR2^-^, CD22^high^*	-	(49, 52)
	Atypical MBC	*TBX21, FCRL5, ITGAX*	T-bet^+^, CD11c^+^	(28, 50)
	Germinal center B	*MS4A1, NEIL1, BCL6, AICDA*	CD20^+^, Bcl6^+^	(45, 53)

### CD4^+^ T cell responses

#### SARS-CoV-2 specific CD4^+^ T cells

**Figure 1:**
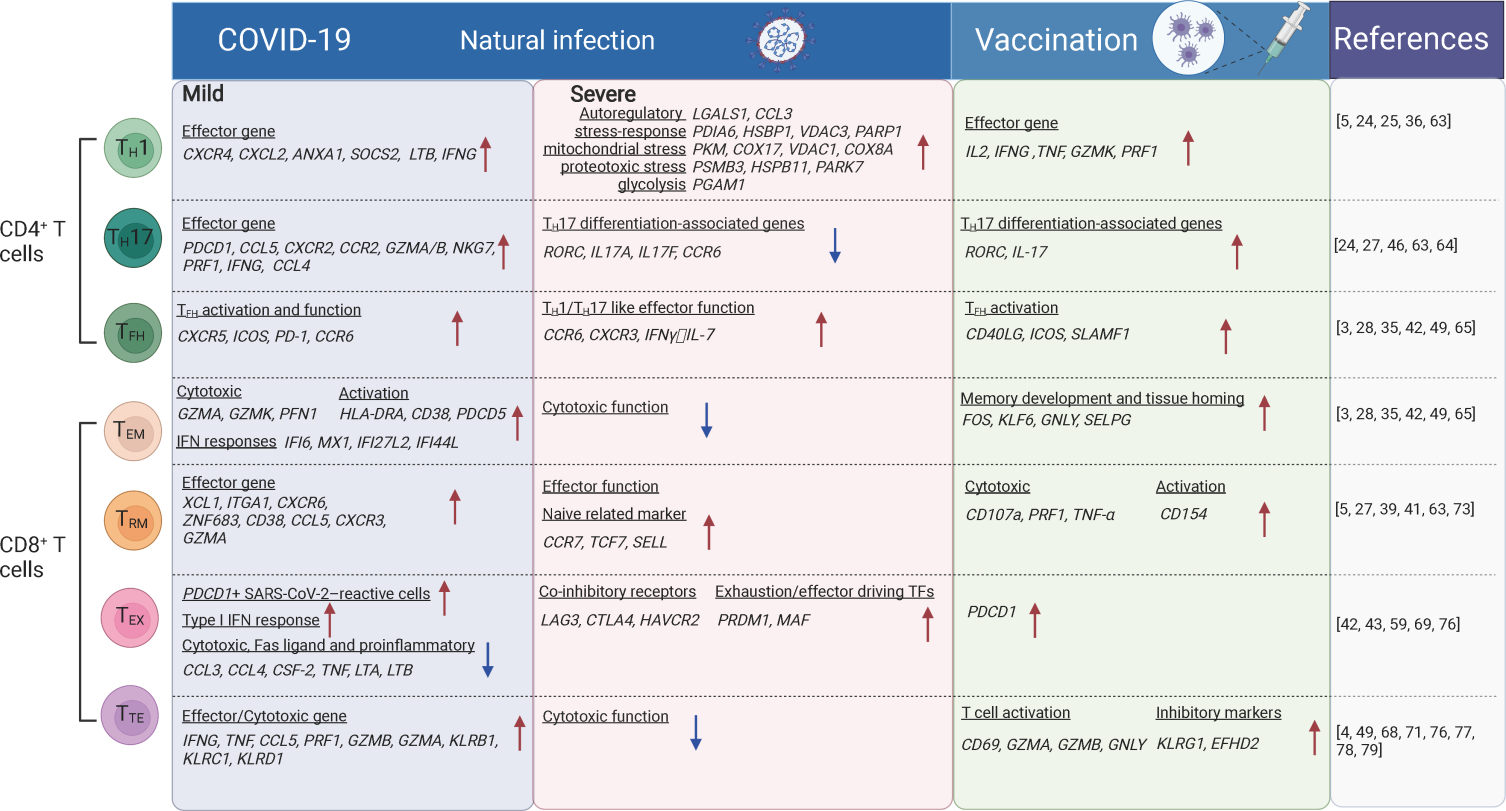
The T cell subsets and their transcriptional changes after SARS-CoV-2 vaccination and infection. The red arrows indicate the genes or pathways that were up-regulated in individuals with COVID-19 or vaccinated compared to the healthy donors, while blue arrows indicate the genes or pathways that were down-regulated. T_H_1, T-helper 1 like cells; T_FH_, T follicular helper cells; T_H_17, T-helper 17 like cells; T_EM_, effector memory; T_TE_, cytotoxic terminal effector; T_EX_, exhausted-like; T_RM_, tissue-resident memory cells. The figure was created using Biorender.

Using activation-induced markers (AIM) or MHC-II tetramers, circulating SARS-CoV-2 specific CD4^+^ T cell responses have been extensively monitored by FACS (54). SARS-CoV-2 infection and vaccination induce robust SARS-CoV-2 specific CD4^+^ T cell responses that occur prior to the generation of high antibody titers and persist for at least 8-12 months (55). The reactivity of spike-specific CD4^+^ T cells to various SARS-CoV-2 variants including Omicron is well preserved (56, 57). Both FACS and scRNA-seq studies revealed that infection-induced specific (mainly spike-specific) CD4^+^ T cell responses showed memory characteristics, including subsets with T_H_1, T_H_17 and T_FH_ phenotypes (25, 58, 59). Notably, the vaccination-induced spike-specific CD4^+^ T cells were predominately T_H_1-like rather than T_FH_-like phenotype despite the robust and persistent human T_FH_ cell responses in the draining lymph nodes, whereas these SARS-CoV-2-reactive CD4^+^ T cells following infection are enriched for both T_FH_ and T_H_1-like phenotype (60, 61). Moreover, following booster immunization, examined by AIM assay, convalescent individuals mount more robust circulating T_FH_ cell response and generate a distinct spike-specific CD4^+^ T cell population expressing both IFN-γ and IL-10, compared with those uninfected individuals, which was consistent with better recall responses induced by the hybrid immunity (54, 62) (**Figure 1**).

#### T_H_1 and T_H_17 cells

Revealed by CyTOF and scRNA-seq analysis, T_H_1-like and tissue-resident T_H_17-like cells were increased and more clonal expanded in patients with mild COVID-19 (5, 43, 63), suggesting virus reactivity of these cells. T_H_1-like cells from patients with mild COVID-19 exhibited a T_H_1 polarization state, characterized by upregulation of effector marker genes (*PDCD1*, *CCL5*, *CXCR2*, *CCR2*, *GZMA/B*, *NKG7*, *PRF1*, *IFNG*, and *CCL4*) (5, 63) and genes involved in effector function such as *CXCR4*, *CXCL2*, *ANXA1*, *SOCS2* and *LTB* (5). Whereas in critical COVID-19 cases, T_H_1-like cells were predominant with the expression of activation markers (*HLA-DR*, *CXCL13*), auto-reactive markers (*LGALS1*, *CCL3*), stress-response markers (*PDIA6*, *HSBP1*, *VDAC3*, *PARP1*), mitochondrial stress genes (*LDHA*, *PKM*, *COX17*, *VDAC1*, *COX8A*), IL-2 withdrawal-associated stressed genes (*MT1E*, *MT1X*), proteotoxic stress genes (*PSMB3/B6/D4/A7/C3*, *HSPB11*, *PARK7*, *EIF4EBP1*) and glycolysis involved genes (*PGAM1*), suggesting functional dysregulation of theses T_H_1-like cells. Tissue-resident T_H_17-like cells were thought to play protective roles (63, 64), and T_H_17 response was found suppressed in severe COVID-19 cases, along with the reduced expression of typical T_H_17-associated genes including *RORC*, *IL17A*, *IL17F*, and *CCR6* (46). In vaccination recipients characterized by scRNA-seq and FACS, BNT162b2, mRNA-1273, or CoronaVac primarily induces T_H_1-like cell responses. These cells express T_H_1-related cytokines, including IL-2, IFNγ or TNFα, activation markers *CD38* and *HLA-DRA*, cytotoxic molecules *GZMK* and *PRF1*, and transcription factor gene *TCF7* (24, 25, 36) (**Figure 1**). Moreover, T_H_1 cell responses were induced rapidly following infection or vaccination followed by the production of high antibody titers. Robust polyfunctional T_H_17 responses were also observed within CD4^+^ T cell compartments following vaccination (24, 27).

#### T_FH_ cells

Studies of FACS, scRNA-seq, and CITE-seq reported that T_FH_ cells were significantly expanded in asymptomatic and symptomatic cases compared with uninfected people (42, 49), which is indicative of recent antigen encounters and emigration from the germinal center (GC). Spike-specific circulating T_FH_ cells positively correlate with plasma neutralizing activity in COVID-19 patients (65). These cells are enriched for genes involved in type I and type II IFN responses which were notably absent in circulating T_FH_ (cT_FH_) cells from uninfected people (28). Early T_FH_ cell responses correlate with the neutralizing antibody levels after the first dose of vaccination and with the CD8^+^ T cell responses after the second dose of vaccination (3). Significant increase in the expression of signature genes for T_FH_ responses (*CD40LG*, *ICOS*, *SLAMF1*, etc.) and the B cell activation (*TLR7*, *CD80/CD86*, *BCL6*) were observed after vaccination (35) (**Figure 1**). These genes were known to be related to the production of neutralizing antibodies. Moreover, cT_FH_ from vaccinated individuals showed an increased transcriptional signature of TNF-NFκB pathway activation (28), which is linked to improved cT_FH_ survival and robust humoral immune responses in a previous study of influenza vaccination (66).

#### Pathogenic CD4^+^ T cells

Some CD4^+^ T cell subsets are reported to be involved in COVID-19 pathogenesis. Both FACS and scRNA-seq analysis revealed that T_H_2 cells were increased and more clonally expanded in patients with severe COVID-19 (4, 67, 68) and they expressed higher levels of *IL4R* and *MAF* associated with T_H_2 responses. In contrast, lower levels of T_H_2 (*IL4*) responses were observed after vaccination (29). The expression of *GATA3* (T_H_2 transcription factor) was also decreased following SARS-CoV-2 infection and vaccination (24). In addition, ex vivo studies showed an absence of T_H_2 responses to spike peptides (24). IL-22-expressing CD4^+^ T cells (T_H_22-like) were relatively enriched in asymptomatic or mild COVID-19 cases. These cells could be associated with tissue-protective responses which could limit immunopathology (49). Proliferative CD4^+^ T cells expressing the proliferation marker Ki67 were enriched in cases with greater severity. The negative feedback mechanisms induced by FoxP3 in Tregs are altered in the lung, which may also contribute to immunopathology (46).

In summary, T_H_1, tissue-resident T_H_17, and T_FH_ play a protective role in early SARS-CoV-2 infection. These cells could also be elicited by SARS-CoV-2 vaccination. Vaccine-induced spike-specific CD4^+^ T cell responses peaked more rapidly than antibody responses after the two-doses vaccination. By contrast, the unregulated T_H_2 and Treg cell responses may contribute to the immunopathology in severe COVID-19 cases.

### CD8^+^ T cell responses

#### SARS-CoV-2 specific CD8^+^ T cells

SARS-CoV-2 specific CD8^+^ T cells have been extensively characterized in recent single-cell analyses by FACS and scRNA-seq. SARS-CoV-2-specific CD8^+^ T cells show a predominantly effector memory phenotype, in a less terminally differentiated state (69, 70), although terminal effector memory cells and transitional memory cells were also observed for SARS-CoV-2-specific CD8^+^ T cells. These SARS-CoV-2-specific memory CD8^+^ T cells are still present at least one year after the infection (71). ScRNA-seq studies revealed that SARS-CoV-2-specific CD8^+^ T cells during the acute phase of infection expressed genes associated with cytotoxicity (*GZMA*, *GZMK*, and *PFN1*), activation (*HLA-DRA*, *CD38*, and *PDCD5*), proliferation (*MKI67*, *MCM7*, and *NUDC1*), and IFN responses (*IFI6*, *MX1*, *IFI27L2*, and *IFI44L*), compared with that in the recovery phase (71). More SARS-CoV-2 specific CD8^+^ T_EM_ cells are accumulated in asymptomatic/mild cases compared with severe/critical cases. In airways, CD8^+^ T_EM_ cells showed elevated levels of HLA-DR, PD-1 and reduced levels of CD127, suggesting the activated status *in situ* (**Figure 1**). SARS-CoV-2 specific CD8^+^ T_EM_ cells in vaccinees were significantly expanded after the second dose of vaccination examined by scRNA-seq (24). Recently, SARS-CoV-2 specific CD8^+^ T cells from individuals with vaccination, infection and breakthrough infection were extensively profiled using MHC-I multimers and scRNA-seq. It was reported that breakthrough infection mounted vigorous non-spike-specific responses, while vaccination among previously infected individuals led to the expansion of spike-specific T cells and continued differentiation to CCR7^-^CD45RA^+^ phenotype. More importantly, TCR analysis of SARS-CoV-2 reactive CD8^+^ T cells showed no evidence of repertoire narrowing following repeated exposure (72).

#### Tissue-resident CD8^+^ T cells

In addition to SARS-CoV-2-specific CD8^+^ T cells identified in blood, these cells were also readily detected in tonsils and displayed tissue-resident memory phenotypes with high expression of CD103 and CD69 (73). Analyzed by scRNA-seq, scTCR-seq and CITE-seq, CD8^+^ T_RM_ cells showed increased proportion and clonal expansion in the airways of mild COVID-19 cases compared with those in severe/critical cases (5, 39, 41, 63). Along the pseudotime differentiation trajectory, CD8^+^ T_RM_ cells were enriched at the end of the lineage in mild COVID-19 cases, indicating a terminally differentiated phenotype. Moreover, these cells express higher levels of the effector molecules *XCL1*, *ITGAE*, *CXCR6*, and *ZNF683* in mild COVID-19 patients than that in severe cases (5, 41) (**Figure 1**). SARS-CoV-2-specific CD8^+^ T_RM_ cells persisted at least 2 months after viral clearance in the nasal mucosa (74). Accumulation of both CD8^+^ T_EM_ cells and CD8^+^ T_RM_ cells is generally associated with lower disease severity, suggesting that they contribute to better outcomes for COVID-19 cases (49, 75). Interestingly, CD8^+^ T_RM_ cells in the nasal mucosa were expanded up to 12 days post the first and second doses of SARS-CoV-2 mRNA vaccination (27). Thus, CD8^+^ T_RM_ cells are important protective cells against the infection of SARS-CoV-2 and other pathogens.

#### Exhaustion of CD8^+^ T cells

Studies of FACS, scRNA-seq and CITE-seq also revealed that CD8^+^ T_EX_-like cells were phenotypically heterogeneous among patients with different outcomes (42, 43, 76). In addition to the increased expression of the inhibitory checkpoint and cytotoxic markers, CD8^+^ T_EX_-like cells were characterized by proliferation with G2M and S gene scores, especially in critical COVID-19 cases (43). Among the co-inhibitory receptors, *LAG3*, *CTLA4*, and *HAVCR2* were enriched in CD8^+^ T cells from COVID-19 patients that eventually succumbed to the disease, but *PDCD1* and *TIGIT* were enriched in CD8^+^ T cells from COVID-19 patients that eventually recovered and discharged (76). The exhaustion/effector driving TFs (*PRDM1*, *MAF*) were also upregulated in COVID-19 patients that eventually succumbed to the disease. However, there are evidences that the PD-1-expressing SARS-CoV-2-specific CD8^+^ T_EX_-like cells are functional rather than exhausted (69). In addition, SARS-CoV-2 vaccination can also induce PD-1 expression in CD8^+^ T cells (59) (**Figure 1**). Hence, whether CD8^+^ T cell is truly exhausted requires further discussion. Transcriptional signatures alone are not sufficient to indicate whether the cells were more exhausted or merely more activated.

#### Pathogenic CD8^+^ T cells

CyTOF and scRNA-seq studies reported the early upregulation of effector molecules by CD8^+^ T_TE_-cells, typically observed within 7 days from symptoms onset and peaking at 14 days following SARS-CoV-2 infection. CD8^+^ T_TE_-cells are associated with effective SARS-CoV-2 clearance and improved clinical outcomes (68, 77). Since CD8^+^ T_TE_ cells are less enriched in SARS-CoV-2 specific CD8^+^ T cells (71), they may be bystander-activated and contribute to disease control in other settings (77, 78). In the late stage of infection, CD8^+^ T_TE_ cells were enriched in cases with greater severity (49, 79), and they are the most proliferative compartments in COVID-19 patients, especially in severe cases (4). The kinetics of T_TE_-cell responses were prolonged and continued to increase up to day 40 after symptom onset (80). Effector genes expressed by CD8^+^ T_TE_ cells such as *GZMB* drive a clear separation between stable and progressive COVID-19 patients (76). In the patients with moderate COVID-19, CD8^+^ T_TE_ cells showed higher expression of *IFNG*, *TNF*, *CCL5*, *PRF1*, *GZMB*, and *GZMA*, together with genes encoding cytotoxic receptors (*KLRB1*, *KLRC1*, and *KLRD1*) in comparison with severe cases. Besides decreasing cytotoxic function, pro-inflammatory cytokines were also poorly expressed in these cells in patients with severe COVID-19. Hence, peripheral leukocytes are not a major contributor to the putative cytokine storm in COVID-19 cases (81).

Overall, various single-cell technologies revealed that infection elicits more diverse immune phenotypes than vaccination, in which CD4^+^ and CD8^+^ T cells activation increased from day 0 to day 14, peaked at day 28, and decreased from day 28 to day 35 (3, 24, 36). Most vaccines elicited T_H_1-skewed and CD8^+^ T_EM_ responses (24). Early T_FH_ and T_H_1 cell responses correlate with effective neutralizing antibody responses after the first dose, whereas CD8^+^ T_EM_ cell responses were elicited after the second dose (3). Breakthrough infection and booster immunization can induce recall response and continued T cell differentiation.

### B cell responses to SARS-CoV-2

#### B cell subsets

Various B cell subsets were identified using FACS, CyTOF, scRNA-seq, and CITE-seq in COVID-19 patients, including naïve B cells (*TCL1A* ^+^ *SELL* ^+^), atypical memory B cells (atMBC, *ITGAX* ^+^ *FCRL5* ^+^), activated B cells (ABC, CD21^low^ CD27^+^ CD10^-^), memory B cells (MBC, CD21^+^ CD27^+^ CD10^-^), *etc.* According to their class switching states, memory B cells also comprised class-switched (IgD^-^ CD27^+^) and non-class-switched (IgD^+^ CD27^+^) subsets, where the class-switched memory B cells are thought to have undergone affinity maturation through GC reactions. The identification of antibody-secreting cells (ASCs), like plasmablast (PB, *CD79* ^+^ *MS4A1* ^-^ *MKI67* ^+^), ‘active’ plasma cells (*XBP1* ^high^ *MS4A1* ^-^ *PRDM1* ^+^), and ‘terminal’ plasma cells (*XBP1* ^high^ *MS4A1* ^-^ *PRDM1* ^-^ *CCL5* ^high^) were also reported following SARS-CoV-2 infection and vaccination (5, 79, 82) (**Table 2**).

**Figure 2:**
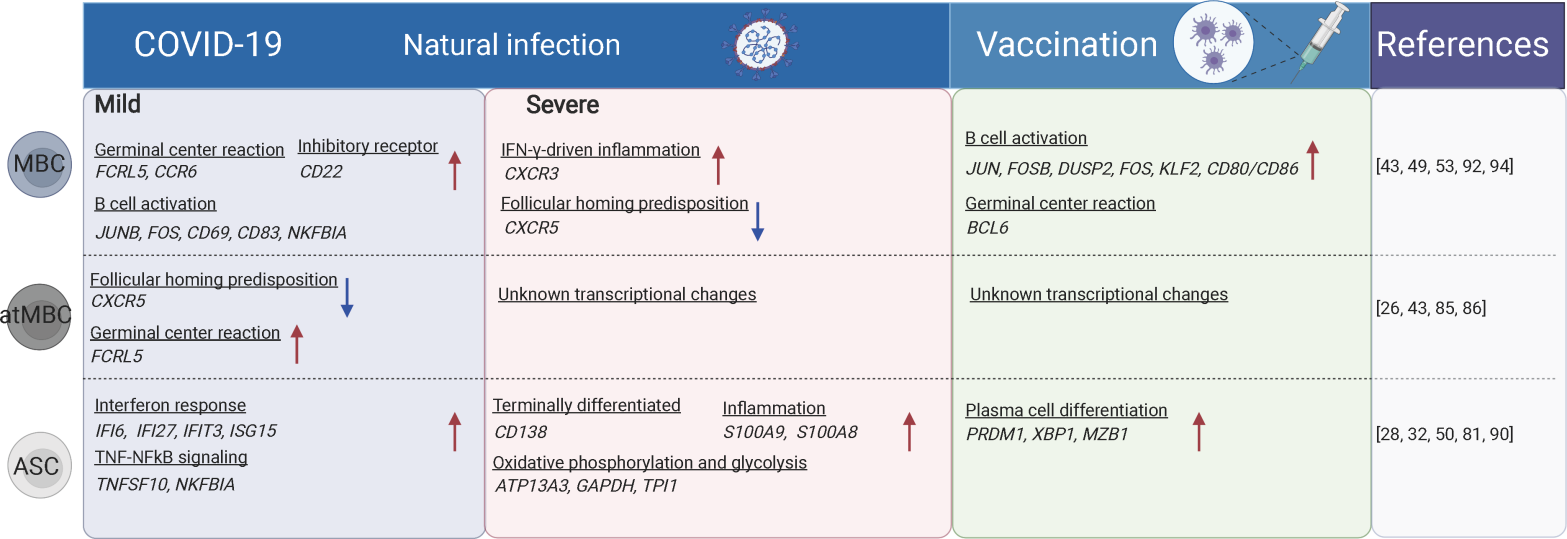
The B cell subsets and their transcriptional changes after SARS-CoV-2 vaccination and infection. The red arrows indicate the genes or pathways that were up-regulated in individuals with COVID-19 or vaccinated compared to the healthy donors, while blue arrows indicate the genes or pathways that were down-regulated. MBC, memory B cells; atMBC, atypical memory B cells; ASC, antibody secreting cells. The figure was created using BioRender.

Previous studies have shown that the proportion of CD19^+^ B cells was increased in severe COVID-19 cases, while transitional B cell subsets were increased in mild/moderate cases. High-dimensional FACS analysis revealed that the proportion of memory B cells was decreased but that of ASCs was increased in severe cases (83). Severe/critical COVID-19 cases also displayed hallmarks of extrafollicular B cell activation which was similar to previously reported in lupus (84) (**Figure 2**). Expansion of atMBC is also a feature of COVID-19 (85). The increased atMBC proportion was positively correlated with COVID-19 severity and decreased to normal levels after recovery (43, 85). AtMBCs in COVID-19 showed impaired proinflammatory effector functions (86). However, spike-specific atMBCs were abundantly produced during secondary immune responses and SARS-CoV-2 infection induces more atMBC than vaccination (26), suggesting that atMBC are functional in these individuals. Therefore, it is unclear whether atMBC is correlated with impaired humoral immune responses during COVID-19. In contrast to atMBC, vaccine-induced ABCs had a similar capacity to bind to specific viral antigens in both SARS-CoV-2-convalescent individuals and naïve subjects (87).

#### Plasma cells

Plasma cells produce antibodies against antigens. Both plasma cells and plasmablasts were more expanded in moderate and severe COVID-19 cases compared to mild cases and uninfected controls (50, 81). Antibodies produced shortly after infection are mostly derived from short-lived plasma cells that are developed through an extrafollicular response. Whereas in the later stage, a smaller population of long-lived plasma cells is generated residing in the bone marrow (88). The PRDM1^-^ plasma cells are characterized by sub-optimal differentiation or activation, which may be defective or even inhibit productive antibody responses in COVID-19. In contrast, PRDM1^+^ plasma cells were supposed to be long-lived populations (89). Plasmablasts are more expanded in hospitalized patients compared to mild/asymptomatic COVID-19 patients (90). The proportions of circulating plasmablasts/plasma cells were positively correlated with the serum levels of TNF, IL-10, and IL-21, which are factors critically involved in B cell differentiation to plasmablasts (42, 90, 91). ScRNA-seq and CITE-seq analyses revealed that these ASCs displayed a high metabolic activity (including oxidative phosphorylation, type I and type II IFN responses, fatty acid metabolism, and mTORC1 signaling), which was reduced following recovery. Meanwhile, memory and naïve B cells showed no significant difference in overall metabolic activity in patients with different disease severity (28). Following vaccination, CD27^+^CD38^bright^ plasmablasts were substantially increased one week later as examined by FACS (32, 50). Besides, plasmablasts from both COVID-19 patients or individuals following SARS-CoV-2 vaccination were enriched for genes involved in IL-6 receptor signaling (JAK/STAT) and inflammatory response, which is consistent with their roles in promoting plasmablast differentiation (28) (**Figure 2**).

#### Germinal center B cells

**Figure 3:**
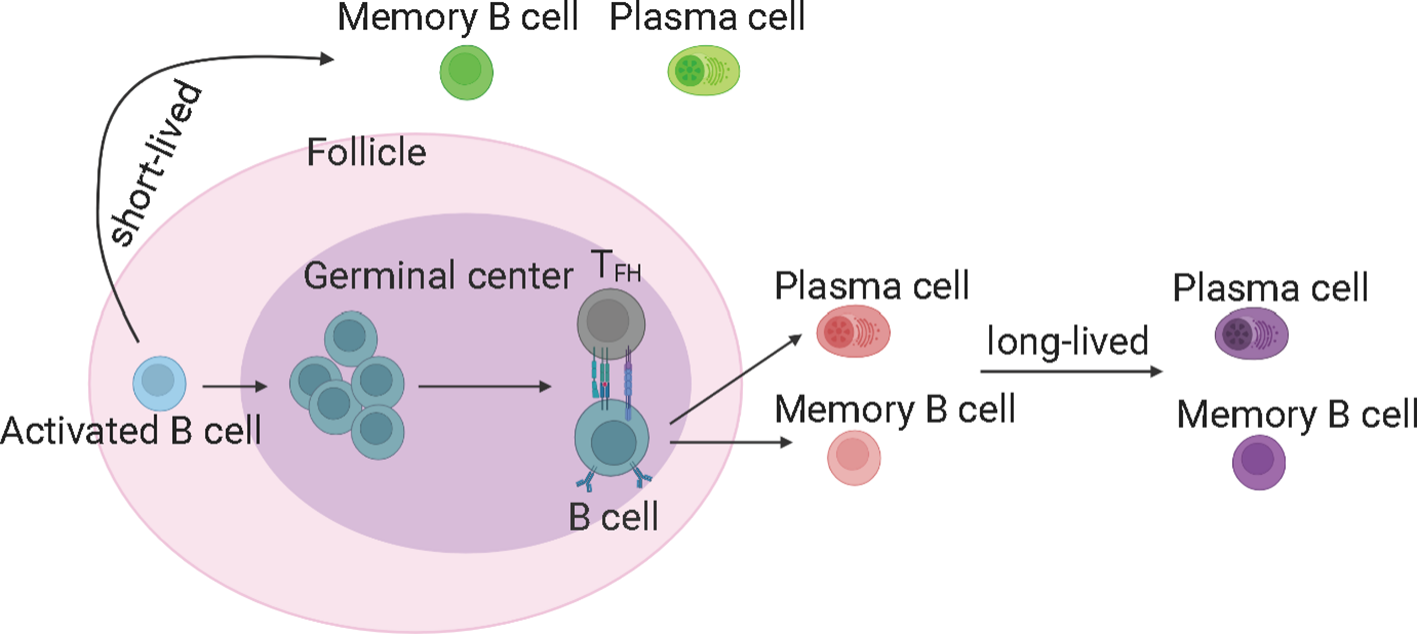
The germinal center reaction and the production of long-lived memory B cells and antibodies. The germinal center derived memory B cells and plasma cells are more stable and long-lived, and able to produce high-affinity antibodies. T_FH_, T follicular helper cells. The figure was created using BioRender.

GC reaction is important and capable of determining the quality of B cell response upon SARS-CoV-2 infection (21). The GC-derived memory B cells and plasma cells are more stable and long-lived (50), and capable of producing high-affinity antibodies (**Figure 3**). The memory B cells can rapidly differentiate into ASCs upon antigen re-encounter. On the contrary, extrafollicular B cell response leads to the production of low-affinity antibodies and wanes rapidly. ScRNA-seq and multi-color immunofluorescence showed that severe COVID-19 cases showed impaired GC reactions than mild cases (43, 92). Thus, the elevated antibody levels and memory B cell responses in severe COVID-19 could be explained by stronger extrafollicular B cell responses. Recently, using fine-needle sampling of lymph nodes and single-cell analyses, some interesting aspects of GC reactions in SARS-CoV-2 infection and vaccination are revealed (21, 93). ScRNA-seq and FACS analysis showed that SARS-CoV-2 vaccination induced a robust specific GC B cell response in the draining lymph nodes, and these GC B cells were maintained for at least 6 months in some individuals, indicating that they are likely to be undergoing further affinity maturation (21, 53, 94). Consistently, it is shown that SARS-CoV-2 memory B cells undergo continued evolution following vaccination or infection, mainly reflected by memory B cells with the increased somatic hypermutation (SHM) and the encoding of the high-affinity and broadly-reactive antibodies (12, 95–97).

#### SARS-CoV-2 specific B cells

The spike-specific class-switched memory B cells and neutralizing antibodies appeared to be stable up to 6 months after infection in COVID-19 patients (22, 98, 99). Vaccination also elicits robust specific B cell responses (88). Single-cell BCR tracing found that the switched memory B cells increased on day 7, and sustained until day 28 (36). In healthy individuals, SARS-CoV-2-specific antibody responses were enhanced upon the second dose of vaccination. Besides, SHM levels of unswitched memory B cells on day 0 are similar to the clonally expanded switched memory cells on days 7-28, suggesting that vaccine-activated unswitched memory B cells can differentiate into switched memory B cells independent of the GC (36, 50, 100, 101). Recently, SARS-CoV-2 specific B cell responses have been comprehensively probed using oligonucleotide-conjugated SARS-CoV-2 proteins at the single-cell resolution. During the early phase of SARS-CoV-2 infection, the pre-existing seasonal coronavirus cross-reactive memory B cells were activated, whereas the SARS-CoV-2-specific B cell clones were gradually accumulated with time and contributed to the majority of memory B cell pool later. The SARS-CoV-2 specific memory B cells maintained the evolution and affinity-mature over several months, by progressively acquiring SHM in GC (21). Over the course of infection and recovery, the number of activated specific B cells decreases while the resting MBCs increase, irrespective of disease severity.

FACS accompanied with biotinylation labeling analysis revealed that spike-specific MBCs were also robustly induced by SARS-CoV-2 vaccination. The induced specific MBCs persist and increase over time after immunization (102). Moreover, SARS-CoV-2 vaccination in recovered individuals induces a significant increase in the antibody response and B cell response, whereas the second dose of vaccine does not induce any further increase in antibody responses. Spike-specific MBCs were not reduced in individuals who had breakthrough infections. In contrast, 5 to 8 months after breakthrough infections, salivary anti-spike IgA levels declined compared with that before SARS-CoV-2 infection. But these salivary anti-spike IgA levels remained significantly higher than in fully vaccinated individuals who never experienced SARS-CoV-2 infection, possibly due to response to a novel antigen (102).

In summary, both SARS-CoV-2 infection and vaccination induce robust humoral immune responses. Severe COVID-19 is usually accompanied by an elevated level of humoral immune responses, likely due to a more robust extrafollicular B cell response. Although the SARS-CoV-2 antibody levels wane rapidly, the SARS-CoV-2 specific memory B cell responses increase over the first several months following the infection and vaccination. Memory B cells progressively acquire somatic mutations in their immunoglobulin heavy variable genes and continue to undergo clonal evolution due to an ongoing GC response. Finally, The booster immunization was effective in increasing neutralizing antibody titers and breadth encoded by vaccine-induced memory B cells against the SARS-CoV-2 variants.

### T cell and B cell immune repertoire

Single-cell based analyses (scTCR-seq and scBCR-seq) have provided key information on the dynamics of TCR and BCR repertoire following SARS-CoV-2 infection or vaccination.

#### TCR repertoire

TCRs are capable of recognizing fragments of antigen as peptides bound to MHC molecules to target virus-infected cells (103). Identification of SARS-CoV-2 T-cell epitopes, generation of MHC multimers, together with single-cell immune repertoire sequencing will facilitate analyses of SARS-CoV-2-specific TCR repertoires (2, 72, 104, 105). The V(D)J genes usage and immune characteristics of TCRs on SARS-CoV-2-specific T cells have been described by several studies, but their function during SARS-CoV-2 infection needs to be further validated.

SARS-CoV-2 immunodominant epitopes may drive the molecular patterns of T cell responses in COVID-19 patients (105). CD8^+^ T-cell repertoire to SARS-CoV-2 was heavily skewed by 70% of all TCR mappings to 5.2% (14/269) of candidate antigen pools (106), suggesting that the T cell response is dominated by recognizing a few specific epitopes linked to distinct HLA types. Indeed, TCRs recognizing HLA-A*01:01-restricted TTDPSFLGRY epitope displayed enrichment for the TRBV27 segment (107). YLQPRTFLL-specific TCRs showed a biased usage of TRAV12-1 (71%) and TRBV7-9 (16%), and RLQSLQTYV-specific TCRs use TRAV13-2 (15%) and TRBV6-5 (25%) more frequently as compared with only 3%-4% gene usage in the control TCRs (108). Although TCRs recognizing HLA-A*02:01-restricted YLQPRTFLL and RLQSLQTYV epitopes were observed in convalescent patients, those TCRs were undetectable or at a very low frequency in the total TCR repertoire of the peripheral blood. It is possible that clones specific to the two antigens are both localized in the tissues, and only a limited number of cells are present in the circulation, which was in accordance with our previous findings that CD8^+^ T_RM_ cells were highly clonally expanded (39, 41). TCRs recognizing HLA-A*11:01-restricted KTFPPTEPK epitope are cross-reactive to various SARS-CoV-2 variants and could confer cytotoxic responses upon encounter with target cells, providing support for developing T-cell epitope incorporating vaccines to prevent continuously emerging SARS-CoV-2 variants (109). By contrast, the antigen-specific TCR repertoire of CD4^+^ T cells was less studied and understood. It was reported that SARS-CoV-2-specific CD4^+^ T cells were increased in patients with severe COVID-19, but these cells displayed low functional avidity and clonality in severe cases than those in mild cases (110), which should be investigated to acquire a better understanding of the underlying mechanisms in future studies.

Various RBD- and spike-specific T cell clones were found from different memory subsets of convalescent COVID-19 patients and individuals receiving SARS-CoV-2 mRNA vaccination. However, there is a limited overlap of TCRs between SARS-CoV-2 infection and vaccination. TRAV29/DV5, TRBV5-1, TRAV29/DV5, and TRBV6-5 are biased in convalescent COVID-19 patients. In contrast, TRAV29/DV5, TRBV11-2, TRAV29/DV5, TRBV7-9, TRAV12-2, and TRBV6-2 are frequently used following vaccination (24). These distinct TCR patterns highlight the differences in the breadth of the epitopes recognized in SARS-CoV-2 infections versus vaccinations (111). These immunodominant epitopes and the TCRs confirmed with functional superiority can better inform next-generation vaccine designs.

#### BCR repertoire

Antibodies, also called immunoglobulins, comprise 5 different classes: IgM, IgD, IgG, IgA, and IgE (112). Different classes of antibodies may form synergy to achieve maximum immune activity against SARS-CoV-2 infection (113). BCR repertoire is the genetic source of neutralizing antibodies. Single-cell analyses have revealed BCR repertoires for total B cells, SARS-CoV-2 specific B cells or plasma cells, facilitating the identification of neutralizing antibodies.

BCR clonality was sharply increased at the early stage of infection and then gradually decreased in the convalescent phase to normal levels (79). BCRs in COVID-19 patients exhibited biased VDJ usage compared with that of healthy controls. The reported SARS-CoV-2 neutralizing antibodies are frequently derived from IGHV3 and IGHV1 family, and more than 40 neutralizing antibodies used IGHV3-53 have been identified. Using single-cell BCR sequencing, it was found that the genes of IGHV3 family including IGHV3-7, IGHV3-15, IGHV3-21, IGHV3-23, and IGHV3-30 were over-represented in COVID-19 patients compared with that in the controls. Besides, the preferred IGKVs were IGKV1-17, IGKV2-28, and IGKV3-15, and the preferred IGLVs were IGLV1-44, IGLV2-8, and IGLV3-27 (51). In contrast, after vaccination, IGHV3-33, IGHV3-43, and IGHV3-49 in the IGHV3 family and IGHV1-69D, IGKV1D-39, and IGLV5-45 were preferentially expanded (24). The cause for different V-gene usage between infection- versus vaccine-induced BCRs is unclear, possibly related to different epitope breadth. Infection elicited B cell clones targeting more epitopes on various SARS-CoV-2 proteins, whereas vaccination mainly induces narrowed antibody responses against S1 and RBD domains.

SHM reduction in COVID-19 patients was consistent with an extrafollicular B cell response mentioned above. RBD-specific clones have been shown to display a low level of SHM (below 5%) during the early stages of infection irrespective of disease severity (114). IgG1, IgG3, and IgA1, and to a lesser extent IgA2 and IgE were dominant isotypes that were rarely mutated or unmutated, indicating that they were derived from an early extrafollicular class switching event. This SHM level increases over time, suggesting an ongoing and persistent GC response.

### Perspectives on adaptive immune responses to COVID-19 at single-cell solution

We have reviewed the broad applications of single-cell analyses in dissecting adaptive immunity in SARS-CoV-2 infection and vaccination. However, the studies of SARS-CoV-2-specific adaptive immunity are still ongoing and many questions remain unresolved.

#### Breadth, diversity, magnitude and lifespan of adaptive immunity

These aspects are the most important characteristics of immune defenses against SARS-CoV-2 infection. The SARS-CoV-2 genome encodes six functional open reading frames (ORFs): replicase (ORF1a/ORF1b), spike (S), envelope (E), membrane (M) and nucleocapsid (N), and seven putative ORFs encoding accessory proteins that are interspersed between the structural genes. More than 2, 000 different SARS-CoV-2-derived epitopes have been curated and reported (IEDB; www.iedb.org), exhibiting great epitope diversity. Structural proteins (S, M, E and N) are predominant targets of T cell and B cell responses (115).

Among these reported epitopes, 95% of reported class II and 98% of class I epitopes were fully conserved in different SARS-CoV-2 variants (Alpha (B.1.1.7), Beta (B.1.351), Gamma (P.1), Mu (B.1.621), Delta (B.1.617.2), and Omicron (B.1.1.529)) (30). For S protein, CD8^+^ T cell epitopes are homogeneously distributed, whereas only a few immunodominant regions were observed for CD4^+^ T cells (116). While the immunodominant epitopes for CD4^+^ and CD8^+^ T cells were noted to be similar in M and N proteins (within 7-101 and 131-213 residues for M protein, and within 31-173 and 201-371 residues for N protein) (105). T cell epitopes in nonstructural proteins show a similar pattern to that of the S protein. CD8^+^ T cell recognition showed more homogeneous patterns, but CD4^+^ T cell epitopes were more evident in nsp3 and nsp12 protein (105).

By contrast, B cell epitopes were prone to immune evasion, especially for the key mutations on spike protein which significantly reduced the neutralization activity of antibodies against SARS-CoV-2 variants (56). B cell epitopes have been extensively mapped for the structural proteins including S, N, M, and E proteins, especially the S. Full-length S or S1 domain which contains RBD were considered a good vaccine antigen as they are the main target to induce neutralizing antibody. Considering the broadly reactive T-cell response versus the waning humoral immunity, we need to incorporate these T-cell and B-cell epitopes outside of spike proteins to develop potentially more effective vaccines.

**Figure 4:**
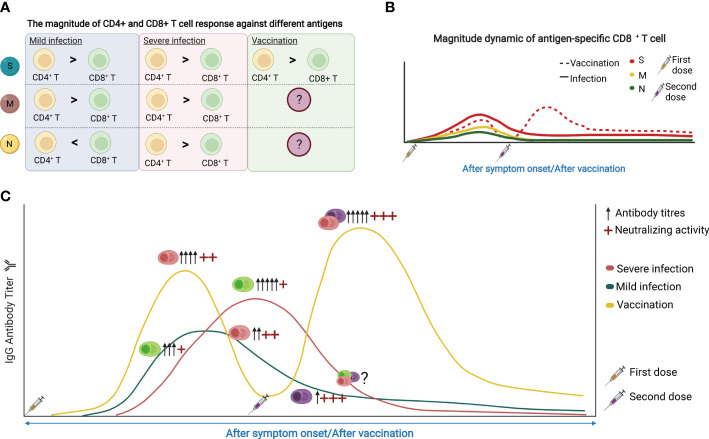
**(A)** The magnitude of CD4^+^ and CD8^+^ T cell response against different antigens. S, spike protein; M, membrane; N, nucleocapsid. **(B)** The dynamic of SARS-CoV-2 reactive CD8^+^ T cell response following SARS-CoV-2 infection and vaccination. **(C)** The dynamic of SARS-CoV-2 reactive antibodies and memory B cells in response to SARS-CoV-2 infection and vaccine. The figure was created using BioRender.

The magnitude of T cell and B cell responses varied when it comes to SARS-CoV-2 infection and vaccination, and is correlated with the disease severity (**Figures 4A, B** **)**. Within T cells, the memory response is skewed toward more CD4^+^ T cell responses than that of CD8^+^ T cells, despite their similar levels immediately after infection (117). Moreover, the S-specific CD4^+^ T cells showed a limited increase after vaccination compared with CD8^+^ T cells (59), while the S-specific CD8^+^ T cell responses after vaccination are weaker. For humoral immunity, vaccination generally induces higher amounts of circulating antibodies compared with infection (**Figure 4C**).

In terms of the duration of memory responses, T cell immunity persists better than antibody responses. However, both SARS-CoV-2 specific memory T cells and SARS-CoV-2 neutralizing antibodies can be detected one year after infection or vaccination (118). Patients with severe COVID-19 showed the delayed engagement of anti-viral CD8^+^ T cell responses compared with mild cases. CD4^+^ T cell responses are more robust than that of CD8^+^ T cells and may even increase in frequency over time, potentially reflecting antigen persistence. However, there was no difference in the magnitude of T-cell responses or neutralizing antibodies in patients with different disease severity one year after infection (118). Vaccines can substantially induce S-specific CD8^+^ T cell responses which peak in most donors at 9-12 days after immunization (59). These S-specific CD8^+^ T cells showed effector memory phenotype with expansion capacity, cytokine production, and degranulation capacity, and remained stable irrespective of vaccine booster (**Figure 4B**). The percentage of B cells increased from day 0-14 but decreased from day 14-28 after immunization. RBD-specific and S1-specific memory B cells may be observed on day 21 and increased gradually over the period of vaccination.

In summary, due to the aforementioned difference in breadth, activity and duration between the SARS-CoV-2 specific T-cell and B-cell responses, and between infection- and vaccination-induced immunity. We propose to improve current vaccination strategies by adding flavors of T-cell components and using heterologous immunization to mimic hybrid immunity to induce more effective, durable and broadly reactive immune responses.

#### High-throughput and accurately dissecting antigen-specific immune responses

Single-cell analyses have revealed various aspects of adaptive immune response to SARS-CoV-2 infections and vaccinations, as described above. However, a deep understanding of how these adaptive immune cells are molecularly regulated remains largely unclear. Single-cell multi-omic technologies such as CITE-seq, scATAC-seq, and scBCR/TCR-seq combined with peptide-MHC multimers will help reveal the underlying regulatory mechanisms driving COVID-19 pathology or prompting long-term protective immunity after vaccination (2, 76). After antigen exposure, immune cells are quickly differentiated into different subtypes to eliminate pathogens through various mechanisms (15, 119). Cell state transition or differentiation between different cell subsets is a multistage and multifactorial process, and single-cell multi-omics can facilitate the exploration of the regulatory mechanism underlying these processes, which is now a challenge due to technological limitations (120, 121). For example, combined scRNA-seq and scATAC-seq can simultaneously profile gene expression and open chromatin from the same cell, enabling deeper characterization of cell types/states and uncovering new gene regulatory mechanisms underlying antigen-specific T/B cell activation, differentiation and memory formation (122). While multi-omic single-cell immune profiling can provide full-length V(D)J sequences for TCRs/BCRs, cell surface protein expression, antigen specificity, and gene expression all from a single cell, allowing clonal tracing of antigen-specific T/B response to infection or vaccination (123). Finally, T and B cells respond to antigen stimulation by metabolic remodeling to execute their functions. Recent advances in single-cell metabolomic analyzing techniques will greatly increase our ability to interrogate these metabolic pathways at single-cell level in antigen-specific lymphocytes (124). Together, the integration of these new technologies can accurately dissect the cellular state transition processes and provide detailed temporal resolution of dynamic changes during infection and vaccination.

Furthermore, identifying and/or isolating antigen-specific T cells or B cells is important not only for the understanding of SARS-CoV-2 induced immune response, but also for providing direct solutions for adoptive immunotherapy (125, 126). However, current protocols for characterizing the immune phenotypes of antigen-specific reactions, including activation-induced marker, degranulation, proliferation, ELISA, ELISpot, intracellular cytokine staining, cytotoxicity, and multimer-based assays, are low-throughput, time-consuming and labor-intensive. New high-throughput single-cell based screening technologies are emerging. LIBRA-seq, an emerging multi-omics application, can simultaneously link antigen specificity with BCR sequencing at single-cell solution (82, 127). Other technologies linking antigen specificity with TCR sequencing will be possibly developed in the future (72). These antigen-specific single-cell sequencings provide effective means to comprehensively characterize the B cell or T cell responses and identify neutralizing antibodies or epitope-specific TCRs.

#### Spatial-temporal immune response

The tissue-resident immunity fights against pathogens at the front line of the host (128–130). Upon SARS-CoV-2 infection, circulating T cells need to be quickly activated and deployed to the site of infection, playing either protective and/or pathogenic roles (131). As the infection resolved, some of these protective effector T cells transformed to long-lived resident T cells, safeguarding the tissues from the potential pathogen re-encounter. In another line of defense, some B cells were initially engaged at the extrafollicular zones where they differentiated into short-lived plasmablasts to provide early antibody responses. Whereas other B cells may undergo GC reactions to produce both long-lived plasma cells and memory B cells (132). Understanding those spatial-temporal processes of SARS-CoV-2 specific T and B cell responses is important but challenging. Although memory T cells and memory B cells were disseminated throughout the body, the source and the distribution of pathogen-specific memory T cell or B cell population have not been systemically elucidated. Single-cell TCR and single-cell BCR sequencing, together with antigen-specific immune cell isolation strategies, can be used to trace the immune cell distribution from the circulation to peripheral tissues, to trace the pathogen-specific memory T (or B) cell turnover, and their differentiation from effector memory phenotype to central memory phenotype over time (4, 28, 72, 123).

In conclusion, the merging multi-omics single-cell technologies will continuously aid the efforts to study adaptive immunity against SARS-CoV-2. The power of these deep immune profiling techniques has already enabled simultaneous analyses of epigenomic, transcriptomic, proteomic, immune repertoire and spatial characteristics of rare populations of SARS-CoV-2 reactive T and B cells. We believe that these advances will greatly prompt both the fundamental and applied studies of SARS-CoV-2 infection and vaccination in the future.

## Author contributions

FQ, SZ, and ZZ wrote and edited the manuscript. FQ and YC created the figures and tables. All authors contributed to the article and approved the submitted version.

## Funding

This study was supported by the National Science Fund for Distinguished Young Scholars (82025022), the Shenzhen Science and Technology Program (ZDSYS20210623091810030), the Shenzhen Bay Funding (2020B1111340074, 2020B1111340075), and the Central Charity Fund of Chinese Academy of Medical Science (2020-PT310-009). The funders had no role in the study design, data collection, data analysis, data interpretation, or writing of the report.

## Conflict of interest

The authors declare that the research was conducted in the absence of any commercial or financial relationships that could be construed as a potential conflict of interest.

## Publisher’s note

All claims expressed in this article are solely those of the authors and do not necessarily represent those of their affiliated organizations, or those of the publisher, the editors and the reviewers. Any product that may be evaluated in this article, or claim that may be made by its manufacturer, is not guaranteed or endorsed by the publisher.
